# Clinical study of suture anchors in the treatment of radial head fractures

**DOI:** 10.1186/s12891-023-06188-1

**Published:** 2023-02-11

**Authors:** Xiao-Nan Li, Yuan-Shen Li, Jun-Lin Chen, Qing-Shan Li, Yan-Hui Suo

**Affiliations:** Department of Sports Injury and Arthroscopy, Handan City Central Hospital, No.15 of Zhong hua Road, Hanshan District, Handan, 056001 China

**Keywords:** Anchor, Internal fixation, Radial head fracture, Comminuted, Treatment

## Abstract

**Background:**

This study aimed to analyze and study the clinical effect of suture anchors in the treatment of radial head fractures (RHFs).

**Methods:**

A total of 11 patients (five male and six female) with RHFs who were treated from March 2016 to June 2021 were included in this study. They were 17–61 (average 38.5) years old. In terms of the Johnston–Mason classification, two cases were type II, seven cases were type III, and two cases were type IV. All patients were treated with open reduction and anchor internal fixation.

**Results:**

All 11 patients were followed up, all incisions healed by first intention, and the duration of follow-up was 14–20 months. The average operation time was 40 ± 15 min. The clinical healing time was 4–6 (average 5) weeks. No patients had any complications, such as traumatic arthritis, malunion, nerve injury, joint stiffness, or incision infection. The clinical effects were evaluated according to the Mayo Elbow Performance Score. The scores of all 11 cases were 90–95, all excellent.

**Conclusion:**

The application of suture anchor internal fixation in the treatment of RHFs has the advantages of accurate reduction, no need for a secondary operation to remove the fixation materials, less trauma, fewer complications, good fracture healing, and good recovery of elbow extension, flexion, and rotation functions.

## Background

Radial head fractures (RHFs) are common in adults, accounting for 1.7–5.4% of fractures [1], including one-third of elbow fractures [2, 3]. Clinical items and processes, including Kirschner wire, micro screws (Herbert screw, absorbable screw, etc.), a micro steel plate system, radial head resection, and radial head replacement, are used in traditional clinical treatment. It is difficult to accurately reset the crushed articular cartilage and have large trauma, complex operation, and high technical requirements. Radial head fracture is an intra-articular fracture, which makes it difficult to reset the dislocation and collapse of the articular surface, which may easily cause joint stiffness and traumatic arthritis, and needs to removed by secondary surgery, which increases the pain and economic burden of patients. From March 2016 to June 2021, all 11 patients with RHFs were treated with suture anchor internal fixation, and the curative effects were satisfactory. The details are reported below.

## Information and methods

### General information

**Fig. 1:**
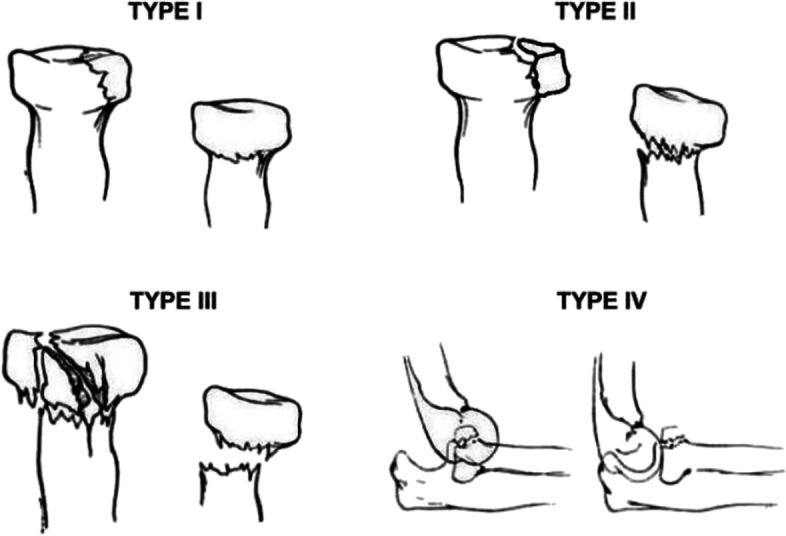
Diagram showing the Johnston–Mason classification for radial head fracture [6, 7]. Type I fractures are non-displaced radial head fractures (or small marginal fractures), also known as “chisel” fractures. Type II fractures are partial articular fractures with displacement > 2 mm. Type III fractures are comminuted fractures involving the entire radial head. Type IV fractures of the radial head involve dislocation of the elbow joint

There were 11 patients enrolled in this study, five males and six females. They were 17–61 (average 38.5) years old. The causes of injury were falls (six cases) and traffic accidents (five cases). In eight cases, the injury was on the left, and in three cases, it was on the right. All were new fractures, and the time from the occurrence of the injury to the operation was 4–8 d. According to the Johnston–Mason classification [4, 5] (Fig. 1), two cases were type II, seven cases were type III, and two cases were type IV. The average operation time was 40 ± 15 min, the clinical healing time was 4–6 (average 5) weeks.

This study was conducted in accordance with the declaration of Helsinki and approved by the Ethics Committee of Handan City Central Hospital. Written informed consent was obtained from all participants.

### Surgical methods

Each operation proceeded as follows. The patient received general anesthesia and was placed in the supine position, abducting the affected limb, and Esmarch’s bandage was applied. The Kocher approach on the outside of the affected elbow was adopted. Starting from behind the lateral epicondyle of the humerus, extending obliquely to the distal end to 3 cm away from the olecranon of the ulna, the skin and subcutaneous tissue were incised. Operation devices entered between the extensor carpi ulnaris and the posterior elbow muscle. The forearm underwent pronation to keep the deep branch of the radial nerve away from the incision, and the fracture of the radial head and neck was explored. The suture anchor (metal 2.8 mm or 3.5 mm or all-suture 4.5 mm) was inserted at the broken end of the fracture towards the medullary cavity or bone cortex of the radial shaft. The anchor was implanted with the tail thread in the direction of the fracture of the radial head and neck, which runs through all the fracture blocks, respectively. Once the articular surface was reduced and the fracture supported, the tail thread of the anchor was tightened, and a knot was made at the neck of the radius. Flexion, extension, and rotation of the elbow joint were completed. When the fracture was stable and the alignment was good, the elbow joint capsule and the lateral collateral ligament were carefully sutured in turn. A C-arm X-ray examination was performed if necessary. After the position was satisfactory, the incision was washed and sutured layer by layer.

## Postoperative care

Broad-spectrum antibiotics were routinely used to prevent incision infection for 48 h after the operation. Postoperative plaster external fixation was given, maintaining the 90° functional position of the elbow flexion. Functional exercise was performed for the distal part of the affected limb from the wrist joint. At 4–5 weeks after the operation, when the X-ray examination showed satisfactory results, the plaster was removed, and active and passive flexion and extension exercises of the elbow joint were started. The X-ray and spiral computed tomography (CT) images were rechecked three, six and 12 months after the operation and the follow-up time was 14–20 months.

## Results

All 11 patients were followed up. All incisions healed by first intention. No wound infection occurred. The clinical healing time was 4–6 (average 5) weeks. The patients were followed up for 14–20 months after the operation, and the elbow extension, flexion, and rotation functions were satisfactory. According to the Mayo Elbow Performance Score (MEPS) [6] (Table 1), comprehensive evaluations were made on the range of motion, pain, stability, and daily activities of the elbow joint, with a full score of 100. The specific evaluation criteria were ≥ 90 for excellent, 75–89 for good, 60–74 points for medium, and < 60 for poor. In this study, the scores of all patients were 90–95 (excellent). All fractures healed well, and the patients had no complications, such as traumatic arthritis, malunion, nerve injury, joint stiffness, or incision infection, and postoperative elbow joint function was shown in Table 2.

**Table 1:** ^[6]^ The Mayo clinic performance index for the elbow.

Function	Point Score
Pain (45 points)	
None	45
Mild	30
Moderate	15
Severe	0
Motion (20 points)	
Are 100 degrees	20
Are 50 to 100 degrees	15
Are 2 degrees	5
Stability (10 points)	
Stable	10
Moderate instability	5
Gross instability	0
Daily function (25 points)	
Combing hair	5
Feeding oneself	5
Hygiene	5
Putting on shirt	5
Putting on shoes	5
Maximum possible total	100

*90 points or more = excellent, 75 to 89 points = good, 60 to 74 points = fair, and less than 60 points = poor

no apparent varus-valgus laxity clinically = Stable

less than 10 degrees of varus-valgus = laxity moderate instability

10 degrees or more of varus-valgus laxity = gross instability

**Table 2:** Postoperative elbow joint function of the patients

Case	1	2	3	4	5	6	7	8	9	10	11
Item											
Postoperative elbow range of motion (elbow flexion)	120°	125°	130°	130°	120°	110°	110°	135°	125°	120°	125°
MAYO motion score	20	20	20	20	20	20	20	20	20	20	20

### Typical case 1

**Fig. 2:**
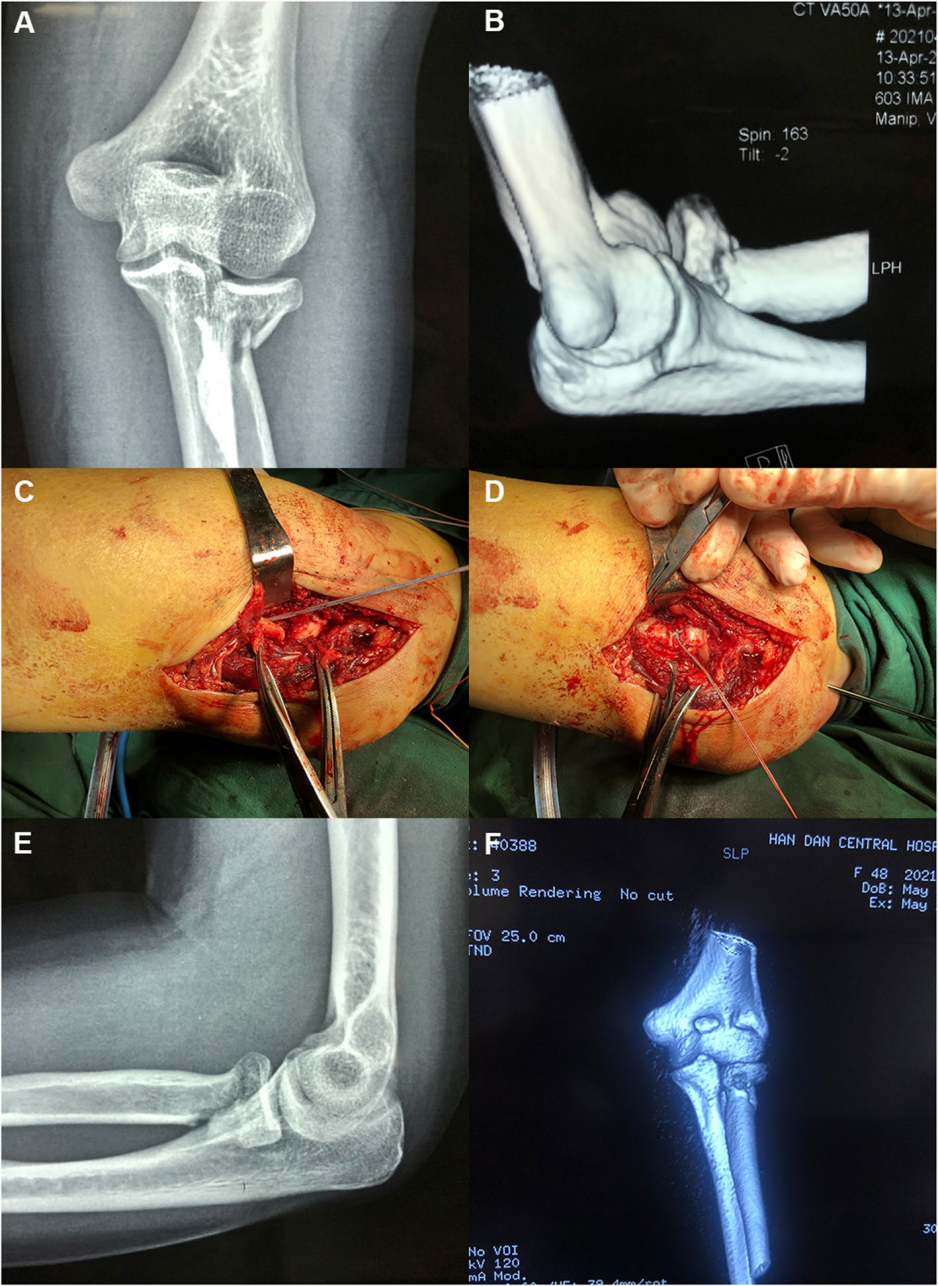
Case 1: Preoperative X-ray (**A**) and spiral CT (**B**) reveal a type III fracture of the left radial head and neck with displacement. During the operation, there is splitting displacement in the junction area of the radial head and neck (**C**), and the articular cartilage at the edge of the radial head is comminuted (**C**, thick arrow). An all-suture anchor (4.5 mm) is implanted into the bone cortex at the distal end of the fracture from inside to outside (**C**, fine arrow). The proximal end of the tail thread of the anchor is folded in a T shape and runs through the osteochondral injury site at the edge of the radial head to the opposite side. After the reduction is satisfactory, the tail thread of the suture anchor is tightened at the neck of the radius and knotted (**D**, thick arrow). A postoperative X-ray (**E**) and CT scan (**F**) revealed that the alignment of the RHF is good

The patient was a 48-year-old female who was admitted to the hospital because of left elbow pain for 7 h caused by a fall injury. A physical examination showed left elbow pain, swelling, and restricted motion. An X-ray and spiral CT revealed Mason type III left radial head and neck fracture displacement (Fig. 2A and B). Open reduction and suture anchor internal fixation of the left RHF were performed 4 d after the injury.

**Fig. 3:**
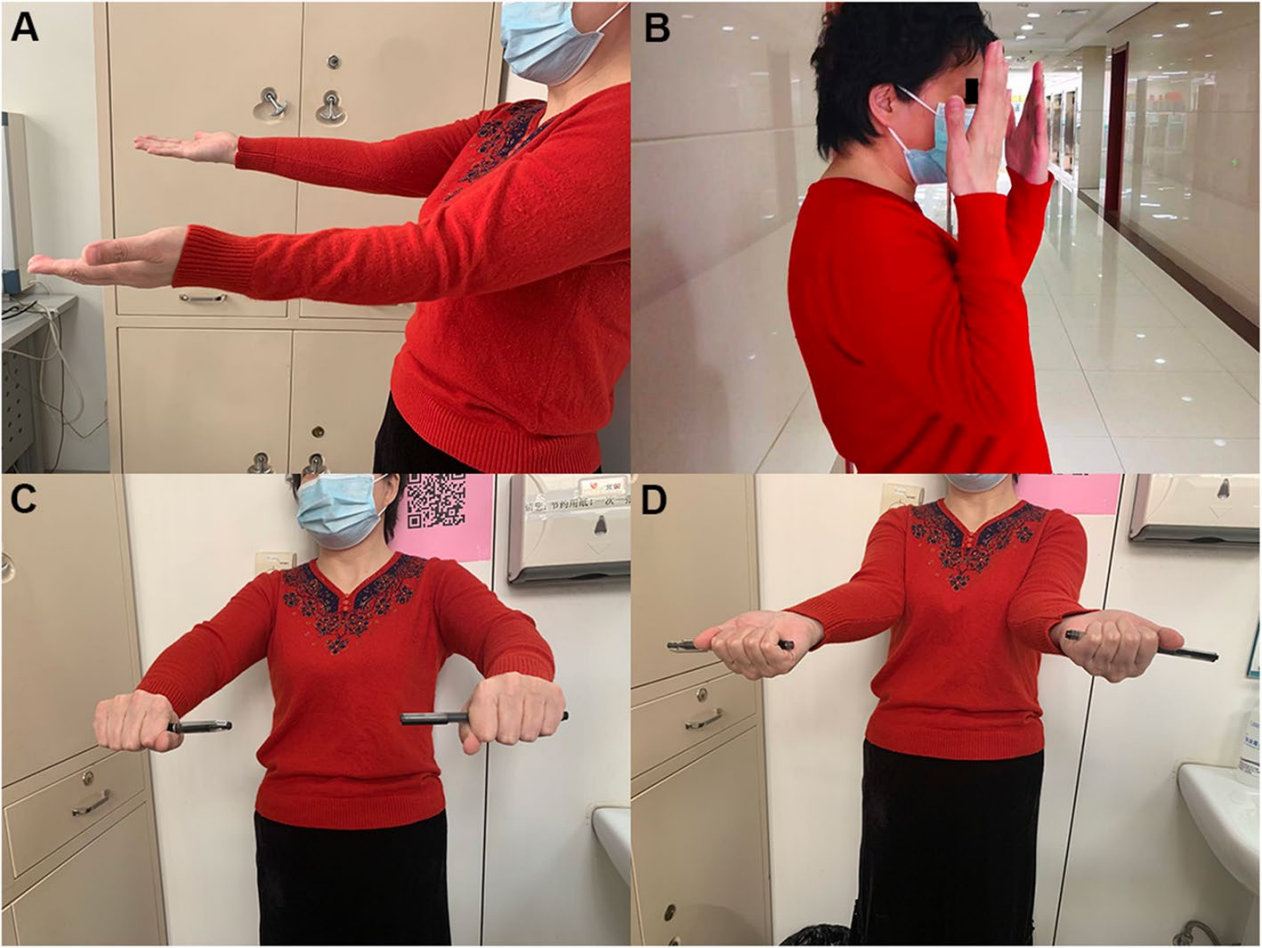
Case 1: The left elbow joint has good extension, flexion, and internal and external rotation functions 10 months after the operation (**A**–**D**)

The Kocher approach, about 6 cm on the outside of the left elbow joint, was adopted. Starting from the back of the lateral epicondyle of the humerus, extending obliquely to the distal end to 3 cm away from the olecranon of the ulna, there was splitting displacement in the junction area of the radial head and neck (Fig. 2C), and the articular cartilage at the edge of the radial head was comminuted (Fig. 2C, thick arrow). An all-suture anchor (4.5 mm) was implanted into the bone cortex at the distal end of the fracture from inside to outside (Fig. 2C, fine arrow). The tail thread of the anchor was folded to the proximal end into a T shape and ran through the osteochondral injury site at the edge of the radial head to the opposite side. After the reduction was adequate, the tail thread of the suture anchor was tightened at the neck of the radius, and a knot was made (Fig. 2D, thick arrow). Flexion, extension, and rotation of the elbow joint were completed, and it could be seen that the fracture alignment was satisfactory and the fracture block was stable. An elbow flexion 90° functional position plaster external fixation was carried out. After 4 weeks, the plaster was removed, and elbow flexion and extension exercises were started. Postoperative X-ray and CT scans showed that the alignment of the RHF was good (Fig. 2 E and F). The patient was followed up 10 months later, at which time the left elbow joint had good extension, flexion, and internal and external rotation functions (Figs. 3A–D), and the MEPS score was 92.

### Typical case 2

**Fig. 4:**
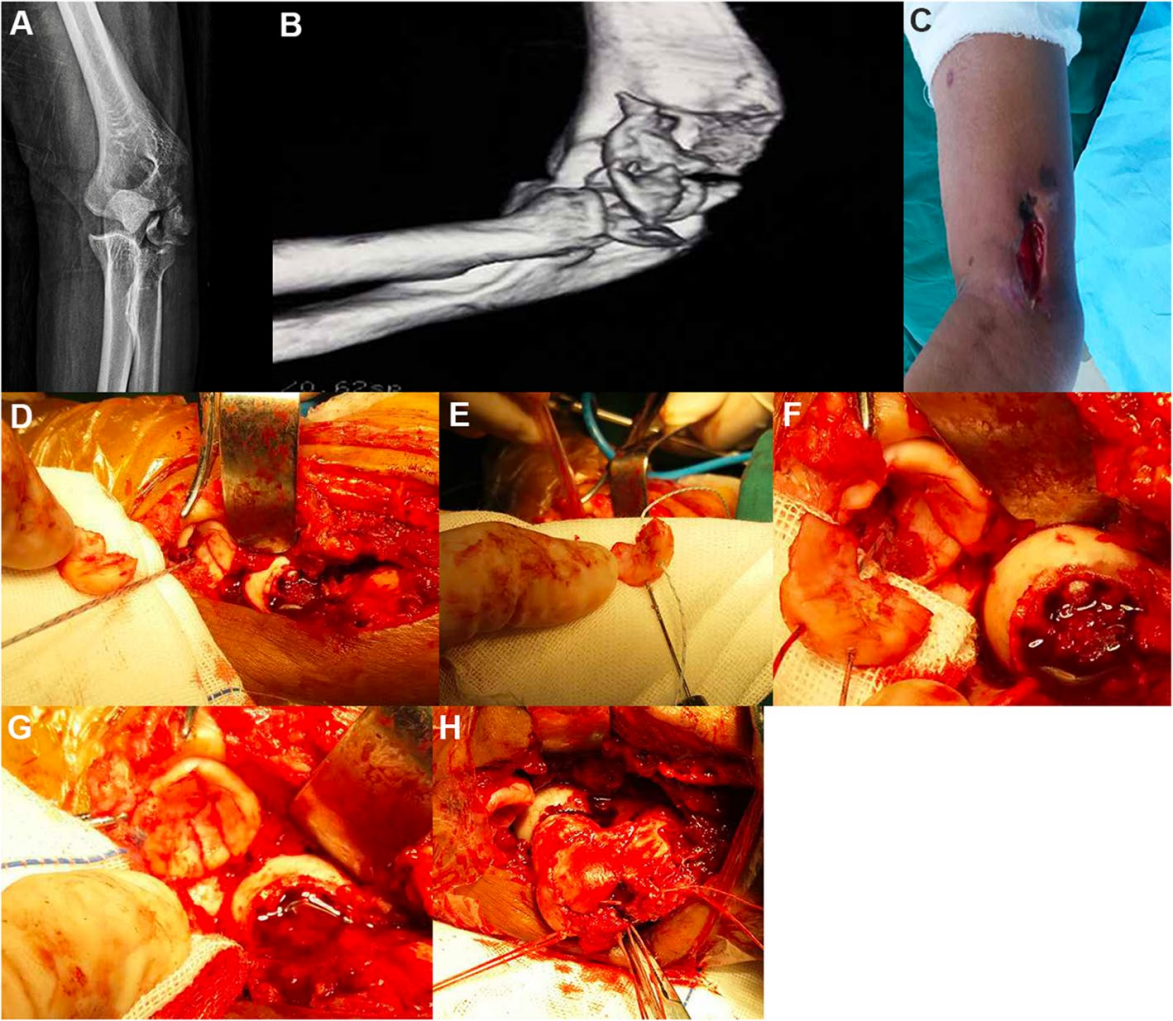
Case 2: A preoperative X-ray (**A**) and spiral CT scan (**B**) reveal a severe comminuted fracture of the radial head (type IV), severe comminution, and dislocation of the humeral capitulum and the trochlea (Bryan and Morrey type IV). During the operation, the incision (**C**) is extended downward from the original wound, with a total length of 12 cm, and the exploration reveals that the articular surface of the radial head is comminuted into four pieces and the subchondral bone has collapsed. A 2.8-mm anchor is fixed in the stronger area of the medullary cavity, the tail thread is led out from the broken end of the fracture (**D**), and the two tail threads of the anchor respectively run in parallel through the freed bone block (**E** and **F**). After the reduction is satisfactory, the two tail threads are tightened and made into a knot at the radial neck (**G**). For the comminuted fractures of the capitulum humeri and the lateral trochlea, similarly, two 3.5-mm anchors are screwed into the bone bed section of the medial semi trochlea, then 8 tail threads of the anchors are run through all freed bone blocks horizontally (**H**). After satisfactory reduction, the threads were tightened and made into knots around the lateral epicondyle of the humerus

The patient was a 43-year-old female who was admitted to the hospital in an emergency 1 h after the injury to her left elbow in a traffic accident. After treatment in other departments for 5 d, the patient was in a stable condition with no surgical contraindication and was transferred to our department. An X-ray and a spiral CT scan revealed a comminuted open fracture of the left radial head with displacement (Mason type IV) (Fig. 4A and B), comminution of the left humeral capitulum and trochlea (Bryan and Morrey type IV). After observing the stability of the whole body, open reduction and anchor internal fixation of the open fracture of the left elbow joint were performed 8 d following the injury.

**Fig. 5:**
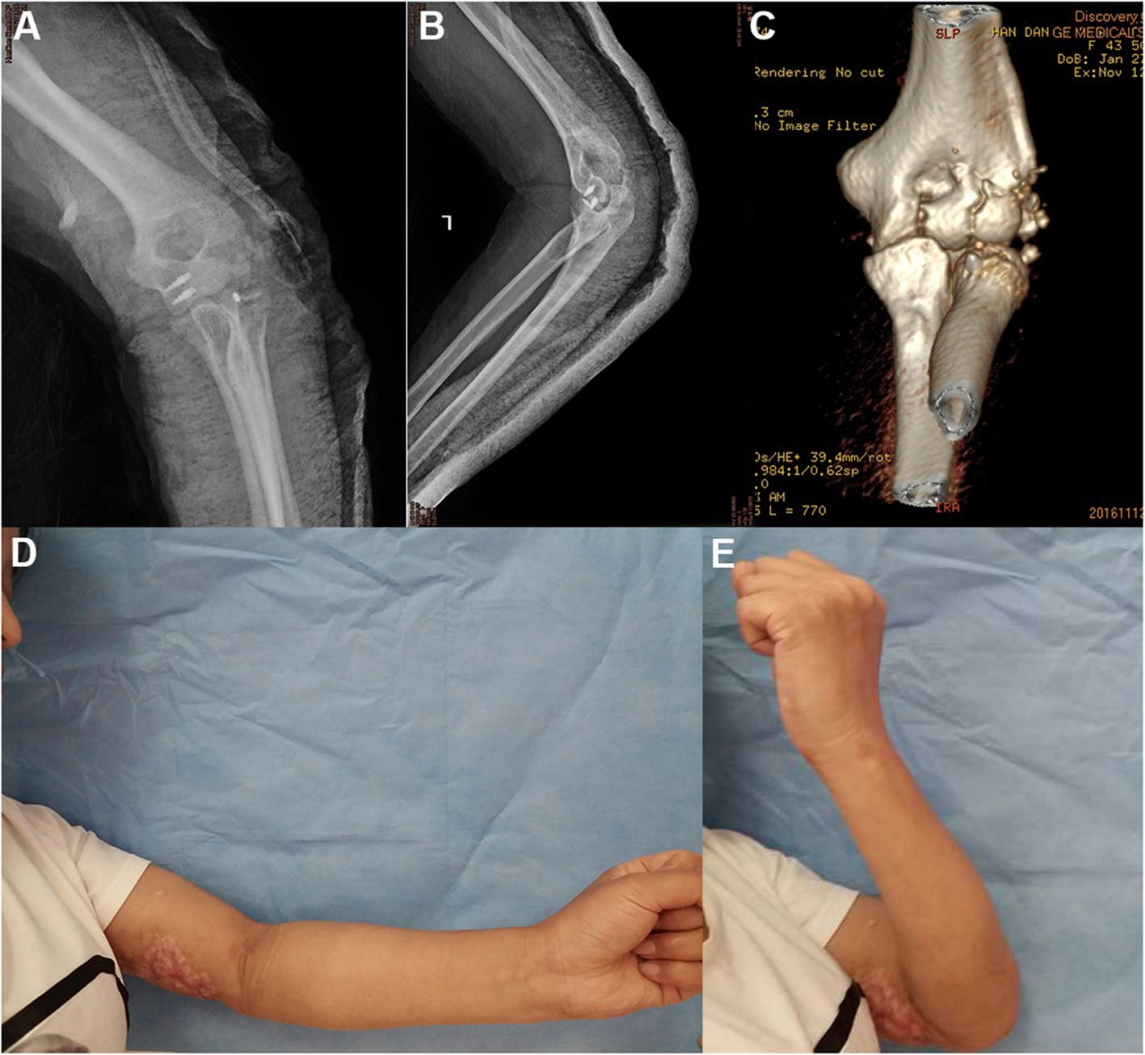
Case 2: A postoperative X-ray (**A** and **B**) and spiral CT scan (**C**) reveal that the radial head and humeral capitulum fractures are in good alignment, and the reduction of the brachial radial joint is satisfactory. The patient is followed up at 10 months after the operation, at which time the extension and flexion functions of the left elbow joint are satisfactory (**D** and **E**)

An incision of about 6 cm was made from the original wound on the outside of the left elbow joint (Fig. 4C), which was extended to the distal Kocher approach, about 12 cm long in total. The skin, subcutaneous tissue, and fascia were incised in turn. The muscle and other soft tissues were seriously contused, the periosteum of the distal humerus was separated, the left radial head was comminuted, the articular surface was fractured into four blocks, and the subchondral bone was collapsed. The small head of the left humerus and the lateral semi trochlea were comminuted into multiple pieces. A 2.8-mm anchor was fixed in the medullary cavity far from the radial neck, one tail thread was led out from the broken end of the fracture (Fig. 4D), and two tail threads of the anchor were respectively passed in parallel through the freed bone blocks (Fig. 4E and F). After a good reduction, the tail threads were tightened and knotted at the radial neck (Fig. 4G). For the comminuted fractures of the capitulum humeri and the lateral trochlea, similarly, two 3.5-mm anchors were screwed into the bone bed section of the medial semi trochlea. The eight tail threads were horizontally run through multiple freed bone blocks of the humeral capitulum and trochlea before being fixed (Fig. 4H). When the fracture alignment was good and the broken end was stable, the tail threads of the anchor were tightened around the lateral epicondyle of the humerus and made into a knot. The joint capsule was sutured, and the lateral ligament and other tissues were repaired. After the operation, elbow flexion 90° external fixation was carried out for five weeks. An X-ray (Fig. 5A and B) and a spiral CT scan (Fig. 5C) were rechecked after the operation, and the results revealed that the alignment of the intra-articular fracture was satisfactory. The patient was followed up 10 months after the operation, at which time the extension and flexion functions of the left elbow joint were good (Fig. 5D and E), and the MEPS of the left elbow joint was 92.

### Typical case 3

**Fig. 6:**
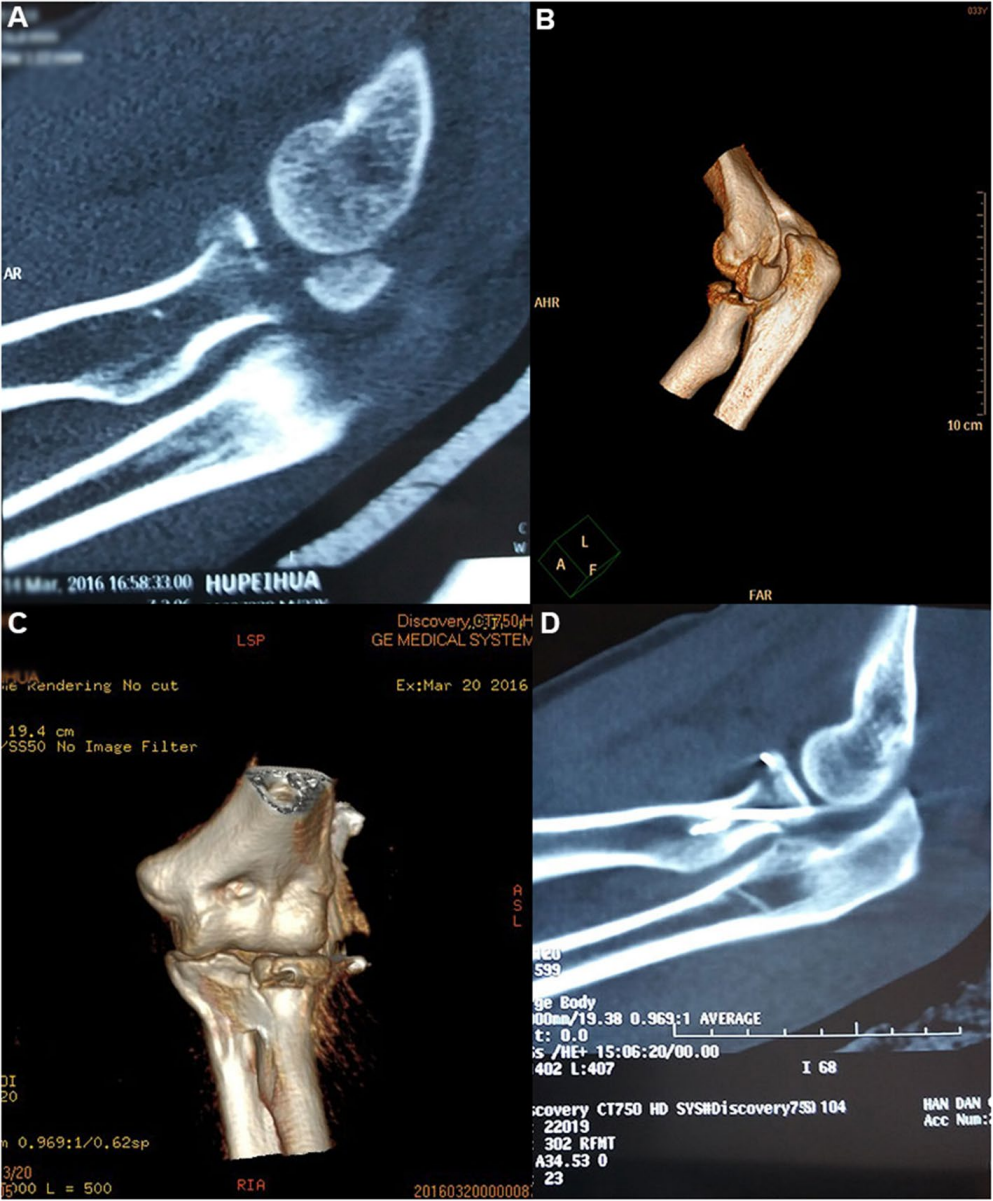
Case 3: A preoperative spiral CT scan reveals a comminuted fracture of the radial head with overturned dislocation (type III) (**A** and **B**). A postoperative spiral CT scan reveals that the reduction of the radial head fracture is good (**C** and **D**)

The patient was a 33-year-old male who was admitted to the hospital due to left elbow pain and limited motion for 3 d caused by a fall injury. The admission examination X-ray and spiral CT scan revealed a left RHF, Mason type III (Fig. 6A and B). Open reduction, suture anchor, and Kirschner wire internal fixation were performed 5 d after the injury.

**Fig. 7:**
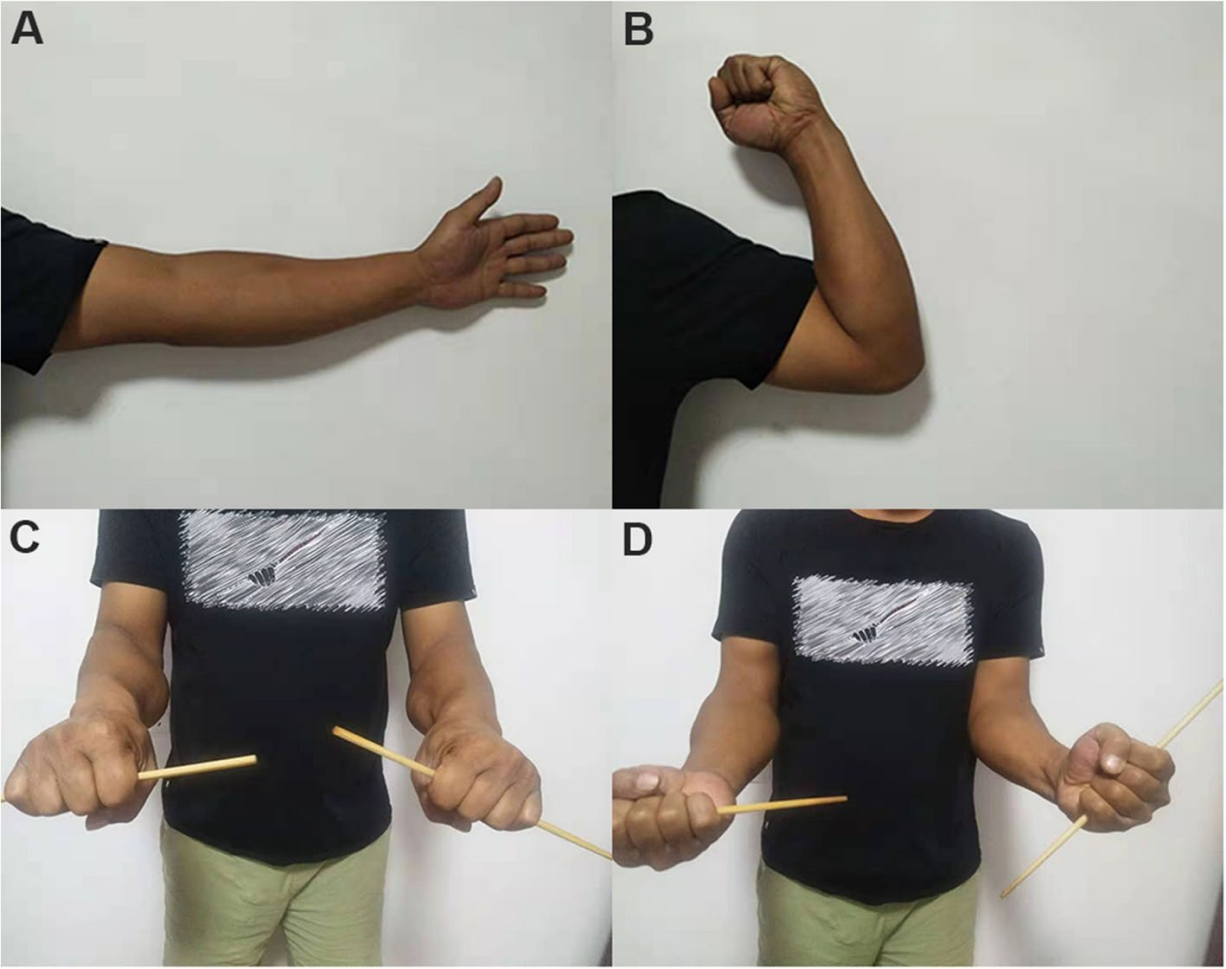
Case 3: The left elbow joint has good extension, flexion, and internal and external rotation functions six months after the operation (**A**–**D**)

An incision of about 6 cm long was made with the Kocher approach on the outside of the left elbow joint, and the exploration showed that the left radial head was seriously comminuted. The larger free bone block was about 2.5 × 2 × 1.5 cm in size, overturned and dislocated. The soft tissue at the broken end of the fracture was removed. A 3.5-mm suture anchor was screwed into the medullary cavity at the distal end of the fracture. The free fracture blocks were drilled horizontally to leave a passage for the anchor tail thread. When all radial head bone blocks were reset satisfactorily and the articular surface was smooth, the tail thread of the anchor was tightened and knotted. Due to the large overturned bone mass, Kirschner wire fixation was carried out for assistance during the operation to enhance stability. After the operation, plaster external fixation at the 90° functional position of elbow flexion was made. A spiral CT scan upon completion (Fig. 6C and D) revealed that the fracture and articular surface were well aligned. The patient was followed up six months later, at which time the patient’s left elbow joint had good extension, flexion, and rotation functions (Figs. 7A–D), and the MEPS score was 95.

## Discussion

The elbow joint consists of the brachial radial joint, the brachial ulnar joint, and the upper ulnar radial joint. The shape of the radial head is like a disk that connects the humeral radial joint with the capitulum of the humerus. The upper ulnar radial joint participates in the rotation of the forearm. The radial head and its surrounding soft tissues are very important in maintaining the stability of the lateral elbow joint. About 60% of the load of the forearm is transmitted through the radial head [7]. After resection of the radial head, the normal load conduction will be destroyed, and there will be a tendency toward valgus. The radial head provides about 30% of the supporting stress, which is the limiting force of the posterolateral, axial, and valgus stress of the elbow joint [8–10]. Therefore, the restoration of the anatomy, shape, and height of the radial head is essential for satisfactory biomechanics of the elbow joint.

Clinically, RHFs are a common type of fracture, accounting for about one-third of elbow fractures. It is seldom caused by direct violence [11]. Mostly, RHFs are caused by indirect violence. The anterior and outer quadrants of the radius are involved in 64% of RHFs [12]. Through high-resolution CT imaging of the radial head, Viveen et al. revealed that the volume fraction and number of trabeculae in the medial quadrant of the radial head were higher than those in the lateral quadrant of the radial head, and there was a difference in the distribution of trabeculae [13], which may be the main explanation for RHFs in the anterolateral quadrant.

Clinically, RHFs belong to intra-articular fractures, especially Mason types II, III, and IV. Conservative treatment often cannot obtain satisfactory anatomical reduction, and if the fracture reduction is poor, complications, such as traumatic arthritis, elbow stiffness, and limited function, occur. The purpose of the operation is to reconstruct the anatomical shape of the radial head, ensure the good flexion, extension, and rotation functions of the elbow joint, and restore the lateral stability of the elbow joint. Strong internal fixation is convenient for early rehabilitation and functional exercise to avoid joint stiffness and traumatic arthritis.

When the displacement of the RHF is > 2 mm, involves more than 30% of the articular surface, and has obvious angular deformity of the radial neck (Mason type II, III, and IV), it needs open reduction and internal fixation. For the treatment of RHFs, the traditional internal fixation is mostly metal implants. Most metal implants must be removed by secondary surgery. When the articular cartilage of the RHF is seriously comminuted, it is often difficult to accurately make the reduction with Kirschner wires and screwa because that may easily result in joint stiffness and traumatic arthritis. Internal fixation with micro plates and screws causes great trauma and is prone to traumatic arthritis, joint stiffness, heterotopic ossification, and elbow pain. In addition. For the micro steel plate, the strict requirements on the placement position increase the difficulty of the operation [14] and are not suitable for all types of comminuted fractures of the radial head [15]. For Mason type III and IV comminuted fractures, radial head resection causes long-term complications, such as elbow instability, traumatic arthritis, elbow pain, limited motion, subluxation of the lower radioulnar joint, and wrist pain. When the RHF is complicated with an injury of the interosseous membrane of the forearm and the dislocation of the lower radioulnar joint, i.e., an Essex–Lopresti injury [16], resection of the radial head causes significant elbow dysfunction [17]. The operation of radial head replacement is complex. In the long term, there will be complications such as prosthesis loosening, joint stiffness, lower ulnar radial joint subluxation, traumatic arthritis, reactive synovitis, and prosthesis wear, which leads to high costs. Its long-term effect has not been verified by follow-up [18–22].

A suture anchor was first used for rotator cuff repair under the shoulder arthroscope, consisting of self tapping screws and high-strength sutures. The anchor suture is made of ultra-high molecular polyethylene fiber, which has the advantages of high strength, easy knotting, and strong flexibility. It will not damage the muscles and bones of patients. The internal fixation is reliable, which is convenient for early functional exercise. The anchor wire material has good histocompatibility. Ettinger et al. [23] believed that the anchor can provide more bone contact and have greater tensile strength. Rickert, Kathleen D and others believed that the biomechanical strength and hardness of screws were better than those of Kirschner wires and screws [24], which could provide reliable fixation effect. Khazen et al. [25] found that multiple anchors can effectively disperse stress and enhance anti rotation torque.

Since 2016, all 11 patients with RHF were treated with suture anchor internal fixation. Its advantages are as follows: (1) The incision is small, and the operation is simple and can be completed without exposing too much soft tissue. (2) The suture anchor is small in design, deeply buried in the bone, needs no secondary operation, and is not limited by the safety area, which is convenient for the operation. (3) The tail thread of the suture anchor can cross each fracture block in multiple directions to form a resultant pressing force converging to the center [26], especially for Mason types II, III, and IV fractures with many blocks. It has its unique advantages. The crushed articular cartilage surface is accurately reduced. The follow-up time of patients in this group is more than one year. X-ray and clinical practice have proved that it can greatly reduce the incidence of postoperative traumatic arthritis. Accurate reduction of the comminuted articular cartilage surface can greatly reduce the incidence of postoperative traumatic arthritis. (4) The internal fixation of the suture anchor is firm, the tail thread has strong toughness, good biocompatibility, and fewer complications, which provides a good environment for fracture healing and facilitates early postoperative rehabilitation.

For 11 cases of radial head fracture in this study, the use of anchor nail treatment is our first initiative. A suture anchor is an ideal internal fixation method for the successful treatment of RHF. Clinically, the department has widely used it in many types of RHF, especially in patients with severe fracture comminution. The reduction operation is simple, the curative effect is remarkable, and there is no need for secondary removal. It is worthy of clinical application and popularization. According to the relevant literature, there is no report on the application of suture anchor internal fixation in the treatment of RHF. Due to the small number of cases included in this study and the limited research data of suture anchors in biomechanics, further observations are needed in the future.

## Data Availability

The datasets generated and/or analysed during the current study are not publicly available due to the lack of an online platform but are available from the corresponding author on reasonable request.

## Abbreviations

RHFs: Radial head fractures
CT: Computed tomography
MEPS: Mayo Elbow Performance Score
